# Association between serum cholinesterase and the prevalence of atrial fibrillation in Chinese hypertensive population: a cross-sectional study

**DOI:** 10.1186/s40001-023-01474-z

**Published:** 2023-11-08

**Authors:** Wenjing Xue, Yi Wei, Yuanhui Hu

**Affiliations:** Department of Cardiovascular, Guang’anmen Hospital, China Academy of Chinese Medical Sciences, No. 5 North Line Pavilion, Xicheng District, Beijing, China

**Keywords:** Serum cholinesterase, Atrial fibrillation, Hypertension, Fatty liver, Association

## Abstract

**Background:**

Atrial fibrillation (AF) is a very common arrhythmia with significant incidence rate and mortality. Several studies have shown a notable correlation between non-alcoholic fatty liver disease (NAFLD) and AF. It has been observed that serum cholinesterase (SChE) levels are elevated in individuals with fatty liver. However, the relationship between the SChE index and AF is still unclear. Therefore, the purpose of this study is to explore the association between the SChE index and the prevalence of AF in patients with hypertension.

**Method:**

We collected cross-sectional data from January 2018 to April 2021 based on a retrospective study of cardiovascular disease. A total of 748 patients with hypertension were included, of whom 165 had AF. We used logistic regression models to test the relationship between SChE and the prevalence of AF in hypertensive patients.

**Result:**

In hypertensive patients, the SChE index was significantly associated with AF (OR = 0.723, *P* < 0.001). After adjusting for potential confounding factors, this correlation was still significant (OR = 0.778, *P* < 0.001). The stability of the model was verified by adjusting the variable type of SChE. The data were further stratified according to whether the patient had fatty liver. In the stratified data, the correlation between SChE and atrial fibrillation was still significant (*P* < 0.05).

**Conclusion:**

Our study showed that SChE was significantly negatively correlated with the occurrence of AF in patients with hypertension. And this correlation was not affected by whether the patient had fatty liver.

## Introduction

Atrial fibrillation (AF) is the most prevalent cardiac arrhythmia, affecting approximately 300–1000 million individuals worldwide [1]. Its incidence is positively correlated with age [2]. Epidemiological studies have demonstrated that AF prevalence ranges from 2% in the general population to 10–12% in individuals aged 80 and above [3]. With the global increase in life expectancy and the prolonged survival of individuals with chronic diseases, AF has emerged as one of the most common cardiovascular disorders in the twenty-first century, resulting in a significant rise in healthcare burden [4]. Despite significant progress in understanding AF and its corresponding treatment strategies in the past decade, early screening and diagnosis of AF remain challenging due to its asymptomatic nature and unpredictable onset time. Camm et al. proposed that one-third of all AF patients have asymptomatic AF, a finding that has been subsequently confirmed in other studies [5, 6].

The pathophysiological mechanisms of AF primarily involve electrical remodeling and structural remodeling [7]. Its occurrence and progression are influenced by various factors. These factors can increase the risk of AF to different extents, thereby promoting the initial development and onset of AF alone or in combination [8, 9]. Hypertension (HT) is the most common modifiable risk factor for AF. Studies have demonstrated that hypertensive patients have a 1.7 times higher risk of developing AF compared to individuals with normal blood pressure [10, 11]. Hypertension accounts for more than one-fifth of new cases of AF [12], and the risk of AF increases by 21% for every 20 mmHg increase in systolic blood pressure (SBP) [13, 14]. Therefore, early identification of risk factors for AF is crucial for hypertensive patients. Furthermore, emerging evidence suggests a link between non-alcoholic fatty liver disease (NAFLD) and the risk of AF [15–17]. However, there have been conflicting reports regarding the association between fatty liver disease and AF [18, 19]. Cholinesterase, primarily synthesized by the liver, is an important laboratory indicator for the clinical diagnosis and prediction of NAFLD. Currently, it remains unclear whether serum cholinesterase (SChE) is an independent risk factor for AF. Therefore, the objective of this study is to investigate the association between the SChE index and the risk of AF in a hypertensive population.

## Methods

### Study site and population

The research center and population for this cross-sectional analysis are part of a retrospective study of cardiovascular diseases conducted from September 2021 to April 2022 in the cardiovascular ward of Guang 'anmen Hospital, China Academy of Chinese Medical Sciences, Beijing, China. This study involved the extraction of de-identified electronic medical records of inpatients from the cardiovascular department, covering the period from 2010 to 2022. By conducting clustering analysis on the clinical information, we were able to identify and summarize the characteristic syndromes of patients diagnosed with AF. Guang’anmen Hospital granted ethical approval for this experiment, which strictly complied with the Declaration of Helsinki (2021). We used cross-sectional data collected in the database for the period January 2018 to April 2021, including 748 patients with hypertension, 165 of whom had AF. Inclusion criteria included: (1) HT patients; (2) participants over the age of 18. Exclusion criteria included: (1) participants with acute coronary syndrome, hypertensive crisis, or other diseases that cause significant hemodynamic abnormalities; (2) participants with NYHA cardiac function rating of 3 or above; (3) participants with malignant tumors, infections or hematological diseases; and (4) participants with missing SChE data.

### Data collection procedures

We collected four parts of data. 1. Demographic data: including age, sex, nationality, height, weight, smoking and drinking; 2. Diagnostic information on basic diseases such as hypertension, coronary heart disease (CHD), arrhythmia, diabetes and hyperlipidemia; 3. Cardiac ultrasound data, including Left atrial diameter (LAD), Left ventricular end-diastolic diameter (LVDD), Ejection fraction (EF), Aortic regurgitation (AR), Mitral valve regurgitation (MR), Tricuspid Regurgitation (TR), Left ventricular systolic dysfunction (LVSD), Regional wall motion abnormality (RWMA)0.4. Laboratory test data, including platelet (PLT), Lymphocyte count (LYMPH), Neutrophil count (NEUT), Fasting Blood Glucose (FBG), Uric acid (UA), Triglyceride (TG), High-density lipoprotein cholesterol (HDL-C), Low-density lipoprotein cholesterol (LDL-C), Serum creatinine (Scr), High sensitivity C-reactive protein (hs-CRP), Thyroid stimulating hormone (TSH), serum cholinesterase (SChE).

### Definitions

Hypertension is defined as previously having a HT history or being diagnosed based on office blood pressure (BP), which includes systolic BP ≥ 140 mmHg or diastolic BP ≥ 90 mmHg, or both [20].

Coronary heart disease is defined as previously having a CHD history or confirmed by the International Cardiology guidelines, which includes typical clinical angina manifestations, electrocardiographic changes, and coronary angiography [21].

Arrhythmia is defined as abnormal frequency and/or rhythm of heart beats, including ventricular and supraventricular arrhythmias [22].

Diabetes is defined as previously having a diabetes history or being diagnosed based on blood glucose (blood glucose at any time ≥ 11.1 mmol/L; fasting blood glucose level ≥ 7.0 mmol/L; blood glucose level in 2 h of oral glucose tolerance test ≥ 11.1 mmol/l/L) [24].

Hyperlipidemia, including high total cholesterol (TC), high total triglyceride (TG), mixed high TC and high TG, high low-density lipoprotein cholesterol (LDL-C), and lowered high-density lipoprotein cholesterol (HDL-C) [24].

### Statistical analysis

This study included the clinical data of 784 patients with hypertension in the original database and interpolated the missing data. Among them, height and weight were interpolated according to the mean, and the remaining missing data were filled by multiple interpolation method of the mice function. We stratified participants into two groups based on whether they had atrial fibrillation. And tested the differences between two populations by *χ*2 test, and descriptive parameters were shown as mean (SD) for continuous variables and proportions for categorical variables. Initially, we explored the relationship between SChE levels and AF prevalence through a logistic regression model. Then, we verified the stability of the model by adjusting the SChE variable type.

In addition, we studied the combined effect of fatty liver and SChE on the prevalence of atrial fibrillation. The sample data were divided into two groups according to whether fatty liver was combined. In the stratified data, the correlation between SChE and the prevalence of AF was further explored. In multivariable models, we adjusted for variables indicated as potential confounders: Age, Sex, Nationality, NLR, HCRP, LDL-C, TG, TC, Scr, UA, LYMPH, Pah, RWMA, LVSD, TR, MR, AR, Left ventricular diastolic dysfunction, EF, LVDD, LAD, β-blocker use, Hyperhomocysteinemia, Hyperlipidemia, CHD and Course of HT. A two-sided *P*-value < 0.05 was considered statistically significant. All statistical analyses were performed using R4.2.2.

## Results

### Baseline characteristics of the study subjects

A total of 784 subjects were included in the analysis. The characteristics of subjects are shown in Table 1. The mean age of the participants was 73.38 (SD: 11.69) years old, which included 390 men. In our study population, the average course of hypertension was 13.13 (SD: 11.54) years and 165 (21.05%) participants suffered from AF. Compared with the group with AF, the group without AF consisted of younger people. Moreover, there were significant differences between the two groups in the following aspects, such as β‐blocker use, LAD, EF, Left ventricular diastolic dysfunction, AR, MR, TR, LVSD, Pah, LYMPH, NLR, TG, hs-CRP and SChE.

**Table 1:** Characteristics of the study population

Characteristic	Total (*N* = 784)	Without AF (*N* = 619)	With AF (*N* = 165)	*p*-value
Age (years)	73.38 ± 11.69	72.49 ± 12.30	76.70 ± 8.31	< 0.001
Sex (*N*/%)				0.133
Male	390 (50%)	317 (51%)	73 (44%)	
Female	394 (50%)	302 (49%)	92 (56%)	
Nationality (N/%)				>0.999
Non-Han	36 (4.6%)	28 (4.5%)	8 (4.8%)	
Han	748 (95%)	591 (95%)	157 (95%)	
BMI (kg/m^2^)	25.06 ± 3.23	25.13 ± 3.10	24.80 ± 3.68	0.302
Smoking (*N*/%)				0.503
No	579 (74%)	461 (74%)	118 (72%)	
Yes	205 (26%)	158 (26%)	47 (28%)	
Drinking (N/%)				0.319
No	642 (82%)	502 (81%)	140 (85%)	
Yes	142 (18%)	117 (19%)	25 (15%)	
Course of HT (years)	13.13 ± 11.54	12.38 ± 11.23	15.91 ± 12.26	0.001
HT grade (*N*/%)				0.063
I	96 (12%)	79 (13%)	17 (10%)	
II	245 (31%)	201 (32%)	44 (27%)	
III	443 (57%)	339 (55%)	104 (63%)	
HT stratification (*N*/%)				0.236
Low-Risk	8 (1.0%)	6 (1.0%)	2 (1.2%)	
Mid-risk	27 (3.4%)	24 (3.9%)	3 (1.8%)	
High-risk	62 (7.9%)	51 (8.2%)	11 (6.7%)	
Extremely high-risk	687 (88%)	538 (87%)	149 (90%)	
CHD (N/%)				0.005
No	192 (24%)	166 (27%)	26 (16%)	
Yes	592 (76%)	453 (73%)	139 (84%)	
Hyperlipidemia (*N*/%)				0.033
No	158 (20%)	135 (22%)	23 (14%)	
Yes	626 (80%)	484 (78%)	142 (86%)	
Diabetes (*N*/%)				0.608
No	507 (65%)	397 (64%)	110 (67%)	
Yes	277 (35%)	222 (36%)	55 (33%)	
Fatty liver (*N*/%)				0.260
No	574 (73%)	447 (72%)	127 (77%)	
Yes	210 (27%)	172 (28%)	38 (23%)	
Hyperhomocysteinemia (*N*/%)				0.059
No	606 (77%)	488 (79%)	118 (72%)	
Yes	178 (23%)	131 (21%)	47 (28%)	
β‐blocker use (*N*/%)				< 0.001
No	438 (56%)	375 (61%)	63 (38%)	
Yes	346 (44%)	244 (39%)	102 (62%)	
LAD (mm)	36.96 ± 7.22	35.49 ± 6.03	42.44 ± 8.58	< 0.001
LVDD (mm)	46.08 ± 5.74	45.77 ± 5.58	47.22 ± 6.21	0.007
EF (%)	58.52 ± 8.30	59.32 ± 7.59	55.53 ± 10.03	< 0.001
Left ventricular diastolic dysfunction (*N*/%)				< 0.001
No	181 (23%)	91 (15%)	90 (55%)	
Yes	603 (77%)	528 (85%)	75 (45%)	
AR (*N*/%)				< 0.001
No	464 (59%)	392 (63%)	72 (44%)	
Mild	283 (36%)	206 (33%)	77 (47%)	
Moderate	34 (4.3%)	20 (3.2%)	14 (8.5%)	
Severe	3 (0.4%)	1 (0.2%)	2 (1.2%)	
MR (*N*/%)				< 0.001
No	360 (46%)	316 (51%)	44 (27%)	
Mild	377 (48%)	282 (46%)	95 (58%)	
Moderate	34 (4.3%)	14 (2.3%)	20 (12%)	
Severe	13 (1.7%)	7 (1.1%)	6 (3.6%)	
TR (*N*/%)				< 0.001
No	406 (52%)	371 (60%)	35 (21%)	
Mild	311 (40%)	226 (37%)	85 (52%)	
Moderate	44 (5.6%)	16 (2.6%)	28 (17%)	
Severe	23 (2.9%)	6 (1.0%)	17 (10%)	
LVSD (*N*/%)				< 0.001
No	703 (90%)	569 (92%)	134 (81%)	
Yes	81 (10%)	50 (8.1%)	31 (19%)	
RWMA (*N*/%)				0.014
No	715 (91%)	573 (93%)	142 (86%)	
Yes	69 (8.8%)	46 (7.4%)	23 (14%)	
Pah (*N*/%)				< 0.001
No	634 (81%)	535 (86%)	99 (60%)	
Yes	150 (19%)	84 (14%)	66 (40%)	
LYMPH (*10^9^/L)	1.70 ± 0.73	1.74 ± 0.74	1.52 ± 0.67	< 0.001
NLR	3.13 ± 2.11	2.96 ± 1.85	3.77 ± 2.81	< 0.001
UA (umol/L)	347.03 ± 108.38	340.54 ± 103.28	371.38 ± 122.98	0.003
Scr (umol/L)	80.58 ± 32.35	78.79 ± 29.78	87.29 ± 39.98	0.012
TC (mmol/L)	4.21 ± 1.09	4.27 ± 1.08	3.96 ± 1.11	0.001
TG (mmol/L)	1.54 ± 1.04	1.60 ± 1.09	1.31 ± 0.79	< 0.001
LDL-C (mmol/L)	2.44 ± 0.80	2.49 ± 0.79	2.25 ± 0.79	0.001
hs-CRP (mg/L)	1.99(0.94–5.57)	1.86(0.92–4.49)	2.77(1.32–13.43)	< 0.001
SChE (KU/L)	8.08 ± 2.25	8.38 ± 2.18	6.95 ± 2.14	< 0.001

*BMI* body mass index, *HT* hypertension, *CHD* coronary heart disease, *LAD* left atrial diameter(antero-posterior), *LVDD* left ventricular end-diastolic diameter, *EF* ejection fraction, AR Aortic regurgitation, *MR* Mitral valve regurgitation, TR Tricuspid Regurgitation, *LVSD* Left ventricular systolic dysfunction, *RWMA* Regional wall motion abnormality, Pah Pulmonary artery hypertension, *LYMPH* Lymphocyte count, *NLR* Neutrophil to Lymphocyte ratio, *UA* uric acid, Scr serum creatinine, *TC* serum total cholesterol, *TG* triglyceride, *LDL-C* low-density lipoprotein cholesterol, *hs-CRP* High sensitivity C-reactive protein, *SChE* serum cholinesterase

*P*-value for analysis of comparison between AF patients and non-AF patients

### SChE and prevalence of AF in patients with hypertension

**Fig. 1:**
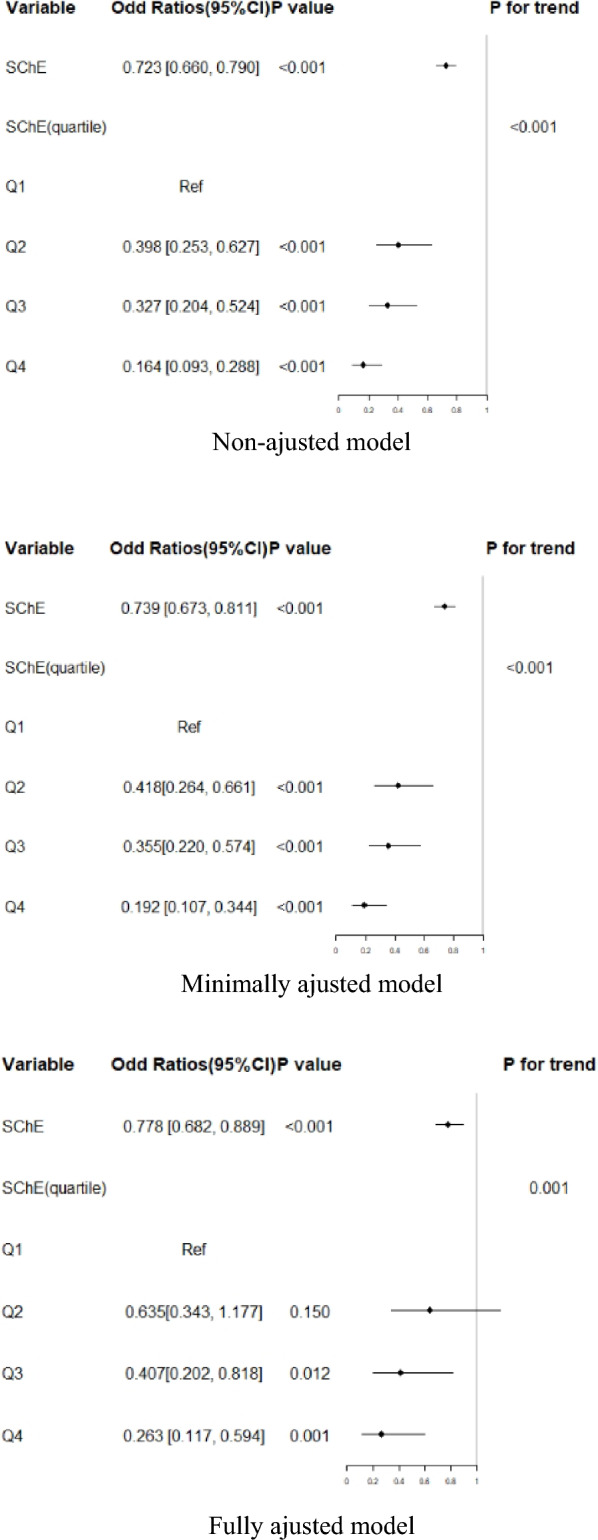
Relationship between SChE and prevalence of AF in different models. ^1^CI Confidence Interval, Ref reference, SChE serum cholinesterase. ^2^Non-ajusted model: we did not adjust other covariants. Minimally adjusted model: we adjusted Age, Sex and Nationality. Fully adjusted model: we adjusted Age, Sex, Nationality,NLR, HCRP, LDL-C, TG, TC, Scr, UA, LYMPH, Pah, RWMA, LVSD, TR, MR, AR, Left ventricular diastolic dysfunction, EF, LVDD, LAD, β-blocker use, Hyperhomocysteinemia, Hyperlipidemia, CHD, Course of HT

We used univariate logistic regression analysis to evaluate the association between SChE and the prevalence of AF. Meanwhile, we also showed the non-adjusted and adjusted models in Fig. 1. In the crude model, SChE showed a negative correlation with AF (OR = 0.723, 95% confidence interval CI 0.660 to 0.790, *P* < 0.001). In the minimally adjusted model (adjusted Age, Sex and Nationality), the result did not have obvious changes (OR = 0.739, 95% CI 0.673 to 0.811], *P* < 0.001). We also detected the same connection in the fully adjusted model (OR = 0.778, 95% CI 0.682 to 0.889, *P* < 0.001). For sensitivity analysis, we handled SChE as a categorical variable (Quartile), and found the same trend as well.

### Association between SChE and prevalence of AF stratified by fatty liver

**Fig. 2:**
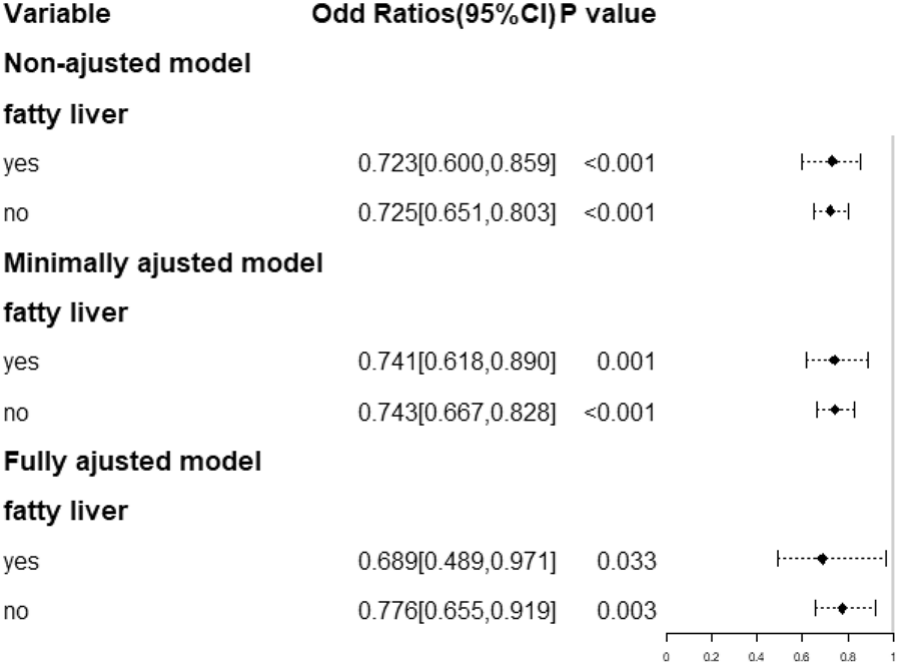
Association between SChE and the prevalence of AF stratified by fatty liver. ^1^CI Confidence Interval, CHESChE serum cholinesterase. ^2^Non-ajusted model: we did not adjust other covariants. Minimally adjusted model: we adjusted Age, Sex and Nationality. Fully adjusted model: we adjusted Age, Sex, Nationality,NLR, HCRP, LDL-C, TG, TC, Scr, UA, LYMPH, Pah, RWMA, LVSD, TR, MR, AR, Left ventricular diastolic dysfunction, EF, LVDD, LAD, β-blocker use, Hyperhomocysteinemia, Hyperlipidemia, CHD, Course of HT

A similar result was observed in the hierarchical analysis, shown in Fig. 2. We also set the non-AF group as the reference group. Then, we stratified the data according to whether participants had fatty liver. SChE was considered to have an association with the prevalence of AF (OR = 0.689, 95% CI 0.489 to 0.971) in the group of participants who had fatty liver. The OR value of participants without fatty liver was 0.776 (95% CI 0.655 to 0.919). All results shown above were from an adjusted model, and the trends were mostly unchanged compared with the results before the adjustment.

## Discussion

Hypertension is widely recognized as the predominant risk factor for AF. Studies have demonstrated that individuals with hypertension are 1.7 times more likely to develop AF compared to those with normal blood pressure [10, 11]. Moreover, patients who have experienced prolonged high blood pressure or inadequate blood pressure management are at a heightened risk of experiencing complications associated with AF, notably stroke, heart failure, and bleeding [25, 26]. Consequently, the early detection of high-risk patients with AF holds significant importance, particularly for individuals with hypertension. As we know, there is a NAFLD association with AF [27]. Liver biopsy is the gold standard for the diagnosis of NAFLD, but its disadvantages such as invasiveness, sampling error, and possible complications limit its clinical application [28]. ALT is a common way to detect NAFLD and assess the severity of liver injury, but its capability to identify NAFLD is doubted [29, 30]. Therefore, the establishment of a more sensitive biomarker to detect NAFLD is necessary. In a large cross-sectional study, SChE was shown to be better markers of fatty liver than ALT [31]. However, the correlation between the SChE index and AF risk in HT patients is still unclear. In this study, we found that SChE levels were significantly associated with AF prevalence in hypertensive patients. In the adjusted model, this relationship still existed. We further stratified the sample data according to whether the participants had fatty liver disease. In the stratified data, the correlation between SChE and AF prevalence was still significant. It showed that SChE may be an independent risk factor for AF.

Serum cholinesterase, also known as pseudo- or butyryl-cholinesterase (BChE), is synthesized by hepatocytes, and its half-life in serum is 11 days [32]. The concentration of SChE is affected by a variety of factors, such as malnutrition, systemic inflammation and liver cell damage [33, 34]. In addition, studies have shown that SChE has a predictive effect on a variety of cardiovascular diseases. There is a positive correlation between high BChE activity and identified cardiovascular risk factors. For example, Goliasch and Alcantara et al. demonstrated that BChE activity values were significantly associated with arterial hypertension [35, 36]. Furthermore, several studies demonstrated an association between BChE activity and metabolic risk factors (such as obesity, hyperlipidemia and diabetes) [37–39]. Interestingly, BChE activity is negatively correlated with cardiovascular mortality and seems to be a predictor of cardiovascular disease prognosis. Low levels of SChE increased the risk of death in patients with acute myocardial infarction [40], acute heart failure, stable coronary heart disease [41], ischemic stroke and other diseases [42]. All of the above conditions may lead to a higher risk of AF. And a study accidentally found a case of cholinesterase deficiency demonstrating sick sinus syndrome (SSS) and short paroxysms of AF associated with a relatively slow ventricular response [43]. Cholinesterase deficiency observed in this patient manifests as the loss of BChE activity. But most patients with silent cholinesterase activity showed no serious arrhythmia, suggesting that some mechanisms compensate for the cholinesterase deficiency [44]. In fact, BChE knockdown has been shown to increase the activity of AChE and other kinases [45]. Currently, most evidence supports that NAFLD may be linked to a slightly higher risk of developing AF [46]. As we all known, SChE is increased in patients with fatty liver [31]. Our research revealed a negative correlation between the SChE index and the risk of AF in hypertensive patients, which differed from our conventional comprehension. This phenomenon can potentially be explained by a new study, which suggested that higher liver stiffness, in particular among those without steatosis, was associated with prevalent atrial fibrillation [47]. This association could be driven by venous congestion instead of fibrogenesis. Therefore, further longitudinal studies with accurate standardized definitions of steatosis and AF are needed to determine strong evidence of the independent association between the two diseases. Based on the currently limited available references, we aim to provide an alternative explanation for this disparity. We hypothesize that the potential mechanism underlying the correlation between the SChE index and AF could be attributed to systemic inflammation. Firstly, our target audience is patients with high blood pressure. Chronic inflammation is involved in the pathogenesis of hypertension [48]. Secondly, it has been shown that the production of BChE and albumin in the liver is coupled, and these biochemical variables may be considered as negative inflammatory reactants whose serum levels are inversely correlated with the increase in the degree of clinical inflammation [49, 50]. In our study, we found that patients with combined AF had lower SChE levels and higher hs-CRP levels compared to patients with only hypertension, which confirmed this point.

For individuals diagnosed with hypertension, it is crucial to promptly identify high-risk patients who may develop atrial fibrillation. This early identification enables early screening, diagnosis, and intervention. Timely and accurate diagnosis of AF can effectively prevent complications, reduce hospitalization related to AF, and lower the mortality rate associated with AF. Although many studies have confirmed that serum biomarkers, echocardiographic markers and specific patterns on brain imaging can predict AF [51–54]. However, to our knowledge, this study is the first to try to determine that SChE is a possible predictive marker of AF in hypertension patients. We found that the SChE index was significantly associated with AF prevalence in hypertensive patients. And the measurement of SChE is easy to obtain in routine blood sampling analysis. Therefore, it represents a cheap and easy-to-obtain AF risk prediction method for hypertensive patients.

Our study has some limitations. First, this was a cross-sectional study and no statements about causality are made. Second, our study had small samples and was single center, which may cause bias. Although we adjusted for confounders in the multivariate analysis, the potential confounders were not completely eliminated. Third, hospitalized patients were typically older and had more underlying diseases. Last, diagnosis of NAFLD was made by ultrasonography rather than liver biopsy, the gold standard technique for detecting fatty liver. And the subjects' drinking history also lacked detailed records, so it was still unclear whether fatty liver was caused by alcohol. In addition, we only included HT patients in this study. Therefore, the suitable population of our findings is limited. Moreover, studies of a large and diverse population should be conducted to further verify. To our knowledge, this is the first study to investigate the association between the SChE index and AF patients with HT.

## Conclusion

In conclusion, our study showed that SChE was significantly negatively correlated with the prevalence of AF in patients with hypertension. And this correlation was not affected by whether patients with fatty liver.

## Data Availability

Supplementary data are available at European Journal of Medical Research online.

## Abbreviations

AF: Atrial fibrillation
NAFLD: Nonalcoholic fatty liver disease
SChE: Serum cholinesterase
SBP: Systolic blood pressure
LAD: Left atrial diameter
LVDD: Left ventricular end-diastolic diameter
EF: Ejection fraction
AR: Aortic regurgitation
MR: Mitral valve regurgitation
TR: Tricuspid regurgitation
LVSD: Left ventricular systolic dysfunction
RWMA: Regional wall motion abnormality
PLT: Platelet
LYMPH: Lymphocyte count
NEUT: Neutrophil count
FBG: Fasting Blood Glucose
UA: Uric acid
TG: Triglyceride
HDL-C: High-density lipoprotein cholesterol
LDL-C: Low-density lipoprotein cholesterol
Scr: Serum creatinine
hs-CRP: High sensitivity C-reactive protein
TSH: Thyroid stimulating hormone
BChE: Butyryl-cholinesterase
SSS: Sick sinus syndrome
